# Three-Dimensional Organoid-like Co-Culture of Human Endometrial Endothelial and Stromal Cells to Study Endometriosis-Associated Responses

**DOI:** 10.3390/ijms27135645

**Published:** 2026-06-23

**Authors:** Caroline Borgato Guedes, Aline R. Lorenzon, Alexandre U. Borbely, Simone Correa-Silva, Elaine C. Cardoso, Barbara Stefany S. Souza, Elisa Lie Matsumura, Tatiana C. de Souza Bonetti, Thais Sanches Domingues, Selma F. Moreira Tsuji, Beatriz Passaro Biscaro, Renata Fioravanti Schaal, Ana Paula Aquino, Eduardo Leme Alves da Motta, Vanessa Morais Freitas, Lidia Hyung Joo Myung, Mauricio S. Abrao, Estela Bevilacqua

**Affiliations:** 1 Department of Cell and Developmental Biology, Institute of Biomedical Sciences, University of São Paulo, Av. Prof. Lineu Prestes, 1524, Butantã, São Paulo 05508-900, SP, Brazil; caroline.b.guedes@gmail.com (C.B.G.); alorenzon@huntington.com.br (A.R.L.); alexandre.borbely@icbs.ufal.br (A.U.B.); enialeccardoso@gmail.com (E.C.C.); babisouza161@gmail.com (B.S.S.S.); elisa.lie@usp.br (E.L.M.); vfreitas@usp.br (V.M.F.); 2 Huntington Medicina Reprodutiva—Eugin Group, São Paulo 04501-000, SP, Brazil; tdomingues@huntington.com.br (T.S.D.); stsuji@huntington.com.br (S.F.M.T.); bpassaro@huntington.com.br (B.P.B.); rschaal@huntington.com.br (R.F.S.); aaquino@huntington.com.br (A.P.A.); emotta@huntington.com.br (E.L.A.d.M.); 3 Cell Biology Laboratory, Federal University of Alagoas, Maceio 57072-900, AL, Brazil; 4 Department of Pediatrics, Faculdade de Medicina, Universidade de São Paulo, São Paulo 05403-000, SP, Brazil; simonecs@alumni.usp.br; 5 Faculdade Israelita de Ciências da Saúde Albert Einstein, São Paulo 05521-200, SP, Brazil; 6 Department of Gynecology, Paulista School of Medicine, Federal University of São Paulo, São Paulo 04039-032, SP, Brazil; tbonetti@unifesp.br; 7 Departamento de Ginecologia, BP—A Beneficência Portuguesa de São Paulo, São Paulo 01323-001, SP, Brazil; lidia.myung@me.com (L.H.J.M.); msabrao@mac.com (M.S.A.); 8 Disciplina de Ginecologia, Departamento de Obstetrícia e Ginecologia, Faculdade de Medicina, Universidade de São Paulo, São Paulo 05403-000, SP, Brazil

**Keywords:** organoid-like 3D co-culture, endothelial cells, endometrial stromal cells, endometriosis, cytokines

## Abstract

Three-dimensional (3D) endothelium–stromal co-cultures were established using human endometrial cells from biopsy of healthy women (*n* = 13) and serum samples from both healthy and endometriotic women (*n* = 5). For 3D construction, stromal cells were mixed with extracellular matrix components, followed by endothelial cell seeding. Morphological analysis confirmed the organization of tissue-like structures. Immunofluorescence and flow cytometry verified the expression of specific stromal and endothelial markers (Cytokeratin, Vimentin, Insulin-like growth factor-binding protein 1, and von Willebrand factor). Cell viability and proliferation increased over time, with minimal cell death. To test functional responsiveness, these co-cultures were exposed to inflammatory serum from endometriotic patients. After 48 h, cytometric bead array showed elevated levels of IL-1β, IL-6, and IL-8 in cultures treated with inflammatory serum, indicating preserved functional activity and responsiveness. By allowing detailed investigation of functional endometrial states within a physiologically relevant cellular network, this approach provides a valuable organoid-like tool to explore conditions such as implantation failure and infertility and to study the cellular interactions underlying reproductive pathologies.

## 1. Introduction

The endometrium functions as a fertility sensor, requiring a specific state to support embryo implantation and early nutrition [1]. Approximately 35–40% of infertility cases are caused by female-specific factors, with endometrial problems often interfering with conception or pregnancy [2,3,4]. These include anatomical issues, inflammation, infections, lifestyle factors, metabolic alterations and immune-related problems [5,6]. Even changes in inflammatory markers in pelvic or peripheral blood can disturb endometrial function, affecting the ability to support implantation and pregnancy [7,8]. Thus, assessments of endometrial physiology are essential for evaluating fertility, both in natural conception and in vitro fertilization [9].

Assessment of complex tissue-interaction systems is a key step in these studies. The objectives of these studies include mimicking certain aspects of the in vivo human implantation environment to allow the investigation of implantation phases as well as understanding uterine physiology and responses to various types of injuries and environmental challenges. Among the several in vitro human models available at present [10,11,12,13,14], three-dimensional (3D) co-culture models offer new possibilities by preserving apicobasal cell polarity, supporting organized spatial cellular interactions, facilitating tissue regeneration, enabling interactions with immune cells, and allowing biochemical gradients that regulate cell communication and signaling. Furthermore, they preserve protein synthesis and secretion domains, similar to those in the in vivo tissue microenvironment, yielding more defined spatial and functional tissue signatures [15,16,17].

The choice of cells used in these studies is also critically important. While cell lines ensure a uniform reproductive response with purer cell populations, primary cells often have a finite lifespan, can be contaminated by neighboring cells, and can introduce variability in experimental results. Experiments with primary cells are also generally more labor-intensive than those with immortalized cell lines. Nevertheless, primary cells offer a unique opportunity to study heterogeneous tissue models, thereby enhancing the relevance of in vivo findings [18,19].

Endometrial stromal cells have been widely used in 3D cultures under different conditions to support long-term survival and function and have improved the understanding of various aspects of uterine physiology [11,20,21]. Notable results have been obtained from studies that utilize organoids to model the embryo–endometrium implantation interface [14,20,21,22,23,24], investigate steroid hormone-driven stromal–epithelial crosstalk [25], examine endometrial senescence [26] and explore endometrial diseases [27]. However, the interactions between tissues and vascular components are an often-overlooked aspect of these studies.

The endothelium plays a central role in maintaining tissue homeostasis and regulating physiological processes [28]. This dynamic interface serves as a physical barrier between circulating blood and surrounding tissues as well as an active signaling platform that integrates biochemical and mechanical stimuli, coordinating communication between the vascular system and other compartments [29,30,31]. Conversely, the endothelial response to circulating factors—such as inflammatory mediators, drugs, metabolites, hormones, and hormone inducers—can create an altered microenvironment that promotes dysfunction in surrounding tissues and, ultimately, contributes to disease [32,33,34]. Understanding this interplay is essential for investigating the formation and maintenance of functional stable tissue networks, especially within both physiological and pathological endometrial contexts.

In this light, we present an experimental organoid-like model that was designed to investigate these cellular relationships and to allow a wide range of functional and pharmacological analyses. The model uses cells isolated from uterine biopsies, focusing on the stromal and endothelial compartments, and reconstructs the tissue in a 3D co-culture system consisting of endothelium-coated uterine stromal cells. We evaluated the morphology and viability of these co-cultures and examined their responsiveness to inflammatory stimuli, using sera from women with endometriosis. Our findings indicated that endometrial cells self-organized into distinct layers, preserving a structural arrangement reminiscent of in vivo endometrial tissue (specifically, the endothelial–stromal cell relationship). Additionally, these cells responded to inflammatory serum by modulating baseline cytokine production, underscoring their functional competence. Since inflammatory conditions can alter serum levels and potentially interfere with endometrial physiology [35,36], our model may offer a novel approach for studying the response of the endometrial environment to such conditions.

## 2. Results

### 2.1. Clinical and Anthropometric Characteristics of Women with and Without Endometriosis

In the analysis of the clinical and anthropometric characteristics of women with and without endometriosis (Table S1), the group without endometriosis, which consisted of 13 participants, had a mean age of 23.5 ± 3.04 years, which was significantly lower (*p* = 0.024) than the mean age of 31.6 ± 5.36 years in the group with endometriosis, which included five participants. The body weight (66.8 ± 10.08 kg in the endometriosis group vs. 60.74 ± 4.23 kg in the control group), height (1.64 ± 0.01 cm vs. 1.61 ± 0.06 cm), and body mass index (BMI) (24.69 ± 4.26 vs. 23.60 ± 2.3) did not differ significantly between the two groups.

When examining the phases of the menstrual cycle, the control group primarily consisted of volunteers in the follicular phase (*n* = 8), followed by those in the ovulatory phase (*n* = 3) and the luteal phase (*n* = 2). In the endometriosis group, four patients were in the follicular phase, while only one was in the ovulatory phase. All women with endometriosis experienced infertility, and the diagnosis and staging of the disease were confirmed by laparoscopy in all cases. Four patients were classified as stage IV and one as stage III. In terms of pain symptoms, three patients reported mild pain, and two reported severe pain.

### 2.2. Distinct Phenotypes Were Observed in the Cells Isolated from Endometrial Biopsies

**Figure 1:**
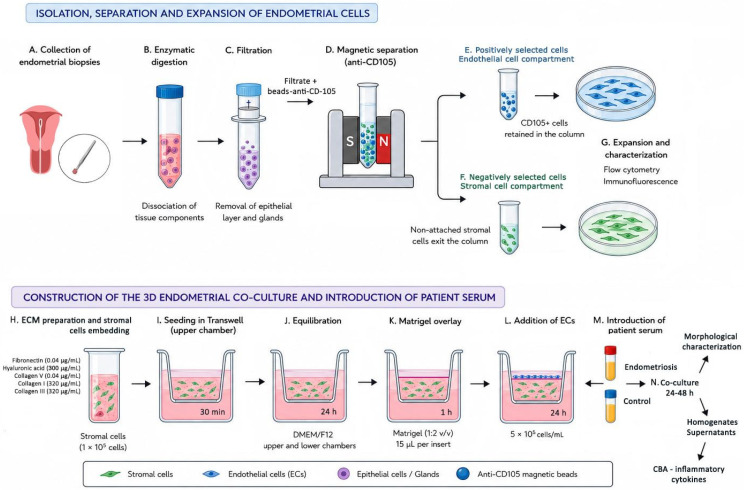
Flowchart illustrating the sequential steps involved in the preparation of endometrial 3D co-cultures. (**A**) The process begins with the collection of endometrial biopsies. (**B**) These samples are subjected to enzymatic digestion to dissociate the cellular tissue components. (**C**) The resulting cells are filtered to remove the epithelial layer and glands. (**D**) A mixture of the filtrate and a solution containing beads bound to anti-CD105 is introduced into a magnetic field. (**E**) The interaction between anti-CD105 and ECs retains these cells in the column. (**F**) Meanwhile, non-attached stromal cells exit the system. (**G**) Positively selected ECs and the negatively selected stromal cells are cultured for expansion and characterized through flow cytometry and immunofluorescence. (**H**) The 3D co-culture system uses an extracellular matrix (ECM) composed of fibronectin, hyaluronic acid, collagen V, collagen I, and collagen III, all diluted in a DMEM/F12. (**I**) Stromal cells (1 × 10^5^) are embedded in this matrix, which is then deposited onto the membrane of the upper chamber of a Transwell. (**J**,**K**) After 24 h of incubation, a Matrigel overlay is applied to the matrix surface. (**L**) Following a 1 h incubation, 5 × 10^5^ ECs in DMEM/F12 are added to the surface. (**M**) After 24 h, the medium is replaced with serum from women with and without endometriosis. (**N**) For morphology analyses, the samples are fixed, embedded in paraffin, and sectioned into serial slices for hematoxylin and eosin (H&E) staining and immunofluorescence. Samples are also processed for ultrastructural analysis. Following model characterization, assays assess the response to the serum from patients with or without endometriosis. Co-culture cells and supernatants are collected after 24–48 h of incubation. Cells are enzymatically isolated and homogenized. The homogenates and the supernatants are analyzed by Cytometric Bead Array (CBA) to quantify cytokine levels. Created in BioRender CC-BA 4.0. https://BioRender.com/9mjo204.

**Figure 2:**
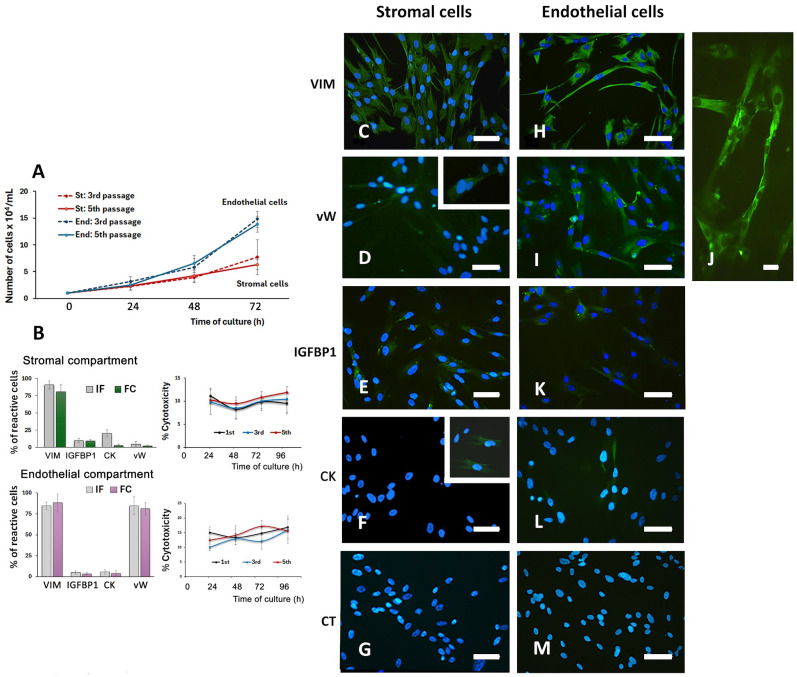
Characterization of endometrial cells isolated by magnetic column. (**A**) Growth curves of stromal (red) and endothelial (blue) cells at passages 3 and 5 show a gradual increase in cell number over the 72 h assay. (**B**) Characterization of the stromal and endothelial compartments showing the percentage of reactive cells for various cellular biomarkers, as determined by immunofluorescence (IF) and flow cytometry (FC). The left panels also display consistently low levels of cell death overtime and across passages 1, 3, and 5 in both compartments. (**C**–**M**) Immunoreactions distinguish stromal cell (**C**–**F**) and endothelial cell compartments (**H**–**L**). Most isolated stromal and endothelial cells are Vimentin-positive (VIM; (**C**,**H**)). Von Willebrand factor (vW) shows strong staining only in the endothelial compartment (**I**,**J**). Conversely, staining for Insulin-like Growth Factor binding Protein (IGFBP1; (**D**,**I**)) and Cytokeratin (CK; (**F**,**L**)) is weak in both compartments. In (**J**), the typical organization of the endothelial cells is shown, with cells contacting their neighbors. (**G**,**M**) Controls for the immune reactions in which the primary is omitted. Immunoreactive cells are stained green (Fluorescein isothiocyanate (FITC)-conjugated antibodies), and nuclei are stained blue with DAPI (4′,6-diamidino-2-phenylindole, Sigma-Aldrich). Except in (**J**), all samples show superimposed DAPI and antibody staining. (bars = 200 μm).

Stromal cells and endothelial cells (ECs) were isolated using immunomagnetic columns and cultured for up to five passages, with each passage treated as a separate group (Figure 1A–G). During this period, both groups exhibited continuous growth (Figure 2A). Cells from each group, cultured for up to 96 h, were characterized by immunofluorescence and flow cytometry, and both methods yielded consistent results (Figure 2B). Lactate dehydrogenase (LDH) assays (Figure 2B) demonstrated consistently low rates of cell death overtime and across passages 1, 3, and 5 in both stromal and endothelial compartments.

#### 2.2.1. Stromal Cells

The cells that were negatively selected from the magnetic column and subsequently cultured showed a fusiform shape and were generally mononucleated, with loose chromatin nuclei and evident nucleoli. Approximately 90.5% + 2.94% of these cells were reactive to Vimentin (Figure 2C), 4.45% + 3.85% to von Willebrand factor (specific to ECs, Figure 2D), 9.68% + 2.32% to IGFBP1 (Insulin-like Growth Factor-Binding Protein 1, the secretory product of decidualized cells, Figure 2E), and 12.3% + 7.5% to Cytokeratin (an intermediate filament typical of epithelial cells of endodermal or ectodermal origin, Figure 2F).

To assess the effectiveness of isolation, the expression profiles of these markers were also realized by flow cytometry (Figure S1) with the following analytical strategy. Initially, cells were selected on the basis of FSC-A vs. FSC-H values (area vs. height) to exclude doublets (cell aggregates) and potential debris present in the samples (first gate; Figure S1A). Subsequently, a second gate was employed to select the cells of interest on the basis of their size and granularity (FSC-A vs. SSC-A, respectively; Figure S1B). The final gate was defined using the viability marker (V510), wherein viable cells were identified as those negatively stained for V510. Stromal cells exhibited a viability of 80.4% (Figure S1C). Assessment of cell isolation efficiency by flow cytometry revealed that 80.2% of stromal cells were positive for Vimentin (Figure S1D), 97.8% were negative for Cytokeratin (Figure S1E), 91% were negative for IGFBP1 (Figure S1F), and 98.4% were negative for von Willebrand factor (Figure S1G).

#### 2.2.2. Endothelial Cells

Cells isolated through CD105 binding (ECs) were elongated, with few but long projections, and often showed a cobblestone shape (Figure 2H). Approximately 84.7% ± 4.56% of these cells were reactive to Vimentin (Figure 2H), 84.8% ± 6.46% to Von Willebrand factor (Figure 2I,J), 90.4% ± 1.24 to CD-105, and 4.98% ± 2.06% and 5.55% ± 2.69% for the IGFBP1 factor (Figure 2K) and Cytokeratin (Figure 2L), respectively. The approach for flow cytometry analysis for ECs was similar to that used for stromal cells (Figure S1H,I). ECs showed a viability of 82.8% (Figure S1J). Evaluation of their isolation efficiency demonstrated that 88.2% expressed Vimentin (Figure S1K), 96% were negative for Cytokeratin (Figure S1L), 97% were negative for IGFBP1 (Figure S1M), and 81.1% were positive for von Willebrand factor (Figure S1N).

### 2.3. Cell Co-Cultures in a 3D System Showed a Tissue-like Arrangement

**Figure 3:**
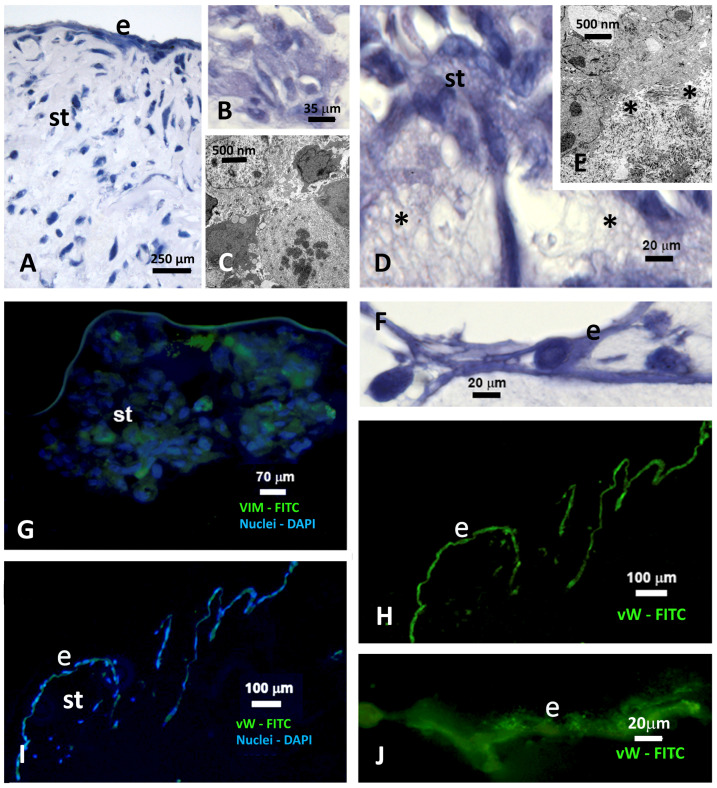
Morphology of 48 h 3D co-cultures of stromal and endothelial cells. (**A**) The reconstructed tissue displays a continuous layer of endothelial cells (e), cells from the endothelial compartment) covering the stromal compartment cells (st), which are dispersed within the extracellular matrix. (**B**,**C**) The images highlight a group of cells (**B**) and mitosis (**C**). (**D**,**E**) Collagen fibrils of the extracellular matrix (*) can be seen in proximity to the cell surfaces. (**F**,**J**) The panels illustrate endothelial cells stained with H&E (**F**) and in sections stained for von Willebrand factor (vW, (**H**–**J**)). (**G**) Immunolocalization of Vimentin (VIM) reveals staining in both stromal and endothelial cells. In the panels (**G**–**J**), green indicates immunoreactive cells, while DAPI stains nuclei blue (**G**–**I**). The images in (**G**,**I**) display an overlay of DAPI and antibody staining.

Co-cultures were maintained for up to 48 h following three-dimensional assembly, after which their morphology was analyzed (Figure 1N). Throughout this period, the cells remained embedded within a substantial ECM and did not retract from the support matrix. Notably, these cells did not exhibit directional organization and had a morphology consistent with that of fibrocytes and fibroblasts (Figure 3A,B,D). Furthermore, the cells demonstrated features typical of viability, including loose nuclear chromatin and prominent nucleoli. Mitosis was also observed in some cells (Figure 3C). At the ultrastructural level, the cells and collagen fibrils in the ECM maintained proximity (Figure 3E). The cells that completely covered this layer demonstrated an epithelioid arrangement (Figure 3A,F) and reacted positively to Vimentin (Figure 3G) and von Willebrand factor (Figure 3H–J). Vimentin was also immunolocalized in cells embedded in the ECM (stromal compartment). The morphology, viability, and biomarker expression patterns remained unchanged regardless of whether control or endometriosis serum was used in the co-cultures and across different cell passages used to assemble the 3D system.

### 2.4. When Exposed to Inflammatory Serum, the 3D Co-Cultures Showed Altered Levels of IL-1β, IL-6, and IL-8

The Interleukin (IL)-6 and IL-8 levels in the cytokine profiles of women with endometriosis (Ep) significantly differed from those in the women without endometriosis (Cp) (Table 1). The increase in IL-1β levels was not significant across the two groups. The levels of IL-12p70, IL-10, and Tumor Necrosis Factor-alpha (TNF-α) were below the respective detection limits (Table 1). Although the groups showed a numerical difference, all IL-12p70 values remained below the assay’s detection limit, rendering quantitative interpretation unreliable. Therefore, this difference was not considered biologically relevant.

The addition of inflammatory serum did not induce morphological changes or cause a significant increase in cytotoxicity rates in the co-cultures in comparison with the findings obtained after treatment with serum from healthy women during the experimental period (Figure S2). In contrast, notable changes in the cytokine profile were detected when the co-cultures were exposed to this serum.

**Figure 4:**
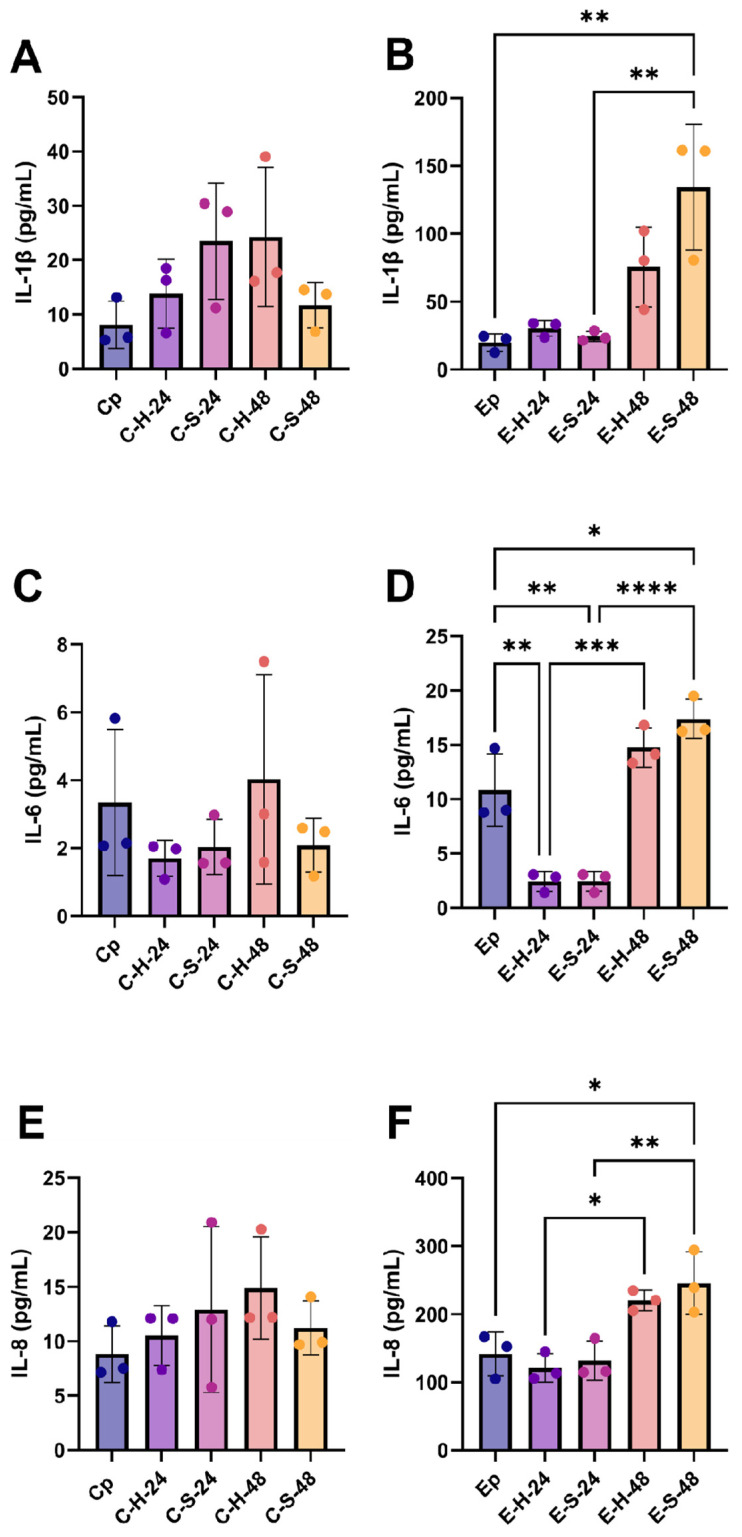
Cytokine profile of 3D-co-cultures incubated with serum from women with endometriosis (E) and without endometriosis (C) for 24 h and 48 h. Ep and Cp represent the values obtained from endometriosis and control patients before cultures. The cytokines IL-1β (**A**,**B**), IL-6 (**C**,**D**), and IL-8 (**E**,**F**) are shown as median ± standard deviation. H: homogenates; S: supernatants. One-way ANOVA followed by Tukey’s post hoc test (*n* = 3). * *p* < 0.05; ** *p* < 0.01; *** *p* < 0.001; **** *p* < 0.0001.

The cytokine profiles of the co-cultures after 24 and 48 h of incubation with control or endometriosis serum were evaluated in cell homogenates and supernatants (Figure 1L–N). The serum from women without endometriosis did not significantly alter the levels of IL-1β, IL-6, and IL-8 in the co-cultures after 48 h of incubation (Table S2), and these values did not differ from those found in patients without endometriosis (Figure 4A,C,E). The levels of IL-12, IL-10, and TNF-α were very low or undetectable; therefore, they were not included in further analysis or discussion.

In contrast, the addition of serum from women with endometriosis to the co-culture significantly increased the cytokine levels after 48 h of incubation. A notable increase in the IL-1β levels was observed in the supernatants over the course of the co-culture (*p* = 0.0022, Figure 4B, Table S2). This increase was also significant in comparison with that in the 48 h control group (*p* < 0.0001; Table S2) and was similarly significant in comparison with that in serum collected from patients with endometriosis (*p* = 0.0016; Figure 4B).

Furthermore, co-cultures treated with endometriotic serum also showed increased levels of IL-6 in the homogenate (*p* = 0.0001) and in the supernatant (*p* < 0.0001) in comparison with the shorter 24 h culture period and 48 h control group (*p* = 0.011 and 0.001, respectively; Figure 4D, Table S1). Specifically, when IL-6 was analyzed against values found in the serum of patients with endometriosis, a significant decrease was observed after 24 h in the homogenates (*p* = 0.0028) and in the supernatants (*p* = 0.0203; Figure 4D), which was followed by a significant increase in the supernatant (*p* = 0.0149) after 48 h of co-culture incubation (Figure 4D).

Similar to IL-6, IL-8 levels were elevated after 48 h of co-culture in both the homogenate (*p* = 0.0169) and supernatants (*p* = 0.0069), in comparison with the measurements taken after 24 h of co-culture (Figure 4F, Table S2). Endometriosis also increased IL-8 levels in the 48 h co-culture supernatants (*p* = 0.0125), in comparison with those in the patients’ sera (Figure 4F). IL-8 levels increased across all serum sources and at all culture times in comparison with their respective control groups (*p* < 0.0001; Figure 4F, Table S2).

## 3. Discussion

In this study, we employed an organoid-like 3D co-culture system consisting of primary human endometrial ECs and fibroblasts. This approach simulated the relationship between the endometrial vascular and stromal compartments. The model used endometrial biopsies and primary cells, rather than the cell lineages or progenitor cells used in classical organoid structures. This choice ensured better proximity to the physiology of in vivo systems. Additionally, storing cells for retesting with samples from the same patient (same genetic heritage) represented a providential advantage.

The methods used to characterize the endothelial and stromal cells in this study were adapted from previous publications [37,38,39,40,41,42], which allowed isolation at comparable rates [37,38]. Characterization by immunofluorescence and flow cytometry analyses yielded results that were well-aligned with the previously obtained findings [37,38]. However, the isolated populations were not entirely pure but showed a predominance of stromal cells and ECs in the two separate compartments, as reported in previous studies [43,44,45]. The presence of relatively few epithelial cells and ECs in the stromal compartment was not an adverse finding, since these components are a natural part of the endometrial stroma in vivo [46]. Based on our morphological approach, the endothelial purity (the proportion of von Willebrand/CD-105-positive cells) in the endothelial compartment was estimated to exceed 80%. Few epithelial cells (positive for Cytokeratin) were detected in the endothelial compartment, as assessed through flow cytometry. However, these cells were not detected on the surface of the co-cultures during immunofluorescence evaluations. One possible explanation for this finding is that cell repositioning may have occurred during interactions between the co-culture compartments.

In the 3D model, tissue stratification resembled in vivo conditions in the endothelial lumen, with the endothelium (von Willebrand-positive cells) completely covering the stromal compartment. In the stromal compartment, the cells were dispersed within the ECM and exhibited characteristics of active cell proliferation in ultrastructural analyses, reinforcing the quality of the model. Studies utilizing the extracellular matrix as a scaffold have highlighted the influence of these components on the morphological and functional behavior of cells cultured in this environment [47,48,49]. In this study, we selected a mixed matrix consisting of type III, I, and V collagens, fibronectin, and hyaluronic acid. All of these components have been previously identified in the endometrium, in varying proportions, depending on the physiological phase of the menstrual cycle and during gestation [50,51].

The co-cultures were standardized using serum from healthy egg donors with no reproductive pathologies and regular menstrual cycles. Serum was collected around the time of ovulation, precluding the need for administration of additional steroids during the short-term culture. Our analysis indicated sustained cell growth and minimal cell death. The 3D model showed healthy cells, demonstrating that the culture conditions and extracellular matrix environment effectively supported cell survival and proliferation. Throughout the development of this model, cells from different passages were used, and the results from the 3D system were consistently replicated.

To investigate the model’s functionality and its capacity to respond to an inflammatory environment, we added serum from women with endometriosis to the co-cultures. Endometriosis is a complex disease influenced by multiple genetic and environmental factors [52,53,54], and affected women exhibit elevated levels of inflammatory cytokines, both systemically and locally in peritoneal fluid [54,55]. CBA assays confirmed the presence of inflammatory factors in the serum of our selected patients. Endometriosis shows wide variation in symptoms, severity, and treatment response and is often described as a heterogeneous disease [52,53,54,55]. Consequently, inflammatory parameters of endometriosis might be inconsistent across experiments. To address this, we used pooled serum from these women as an inflammatory challenge to achieve consistent induction.

Exposure of the co-culture to serum from patients with endometriosis did not alter the cell morphology, biomarker expression, cell viability, or growth. However, it induced a pronounced inflammatory response, as evidenced by a significant increase in IL-1β, IL-6, and IL-8 production. Differences between the cytokine levels originally measured in patients’ serum and those later detected in supernatants and cell homogenates from cultured cells confirm that new cytokine production and release occurred during culture. Notably, IL-6 and IL-8 concentrations in the co-culture supernatants and homogenates were approximately 1.7-fold higher than those in endometriotic patient serum. This comparison indicates that the co-culture itself produced additional IL-6 and IL-8 in response to the patient’s serum, resulting in progressively increasing cytokine levels over time. This represents a modest yet significant inflammatory response.

Low levels of IL-6 have been observed in tissues and monocultures of human endometrial stromal cells, indicating that IL-6 is a consistent component of uterine physiology [56,57]. IL-6 upregulation is also observed in cultures of endometrial cells derived from women with endometriosis [57,58,59]. As also observed in our study, increased IL-6 levels have been identified in the serum and peritoneal fluid of women with endometriosis [54,55,56], indicating a link to the disease [60]. IL-6 plays a dual role: it is associated with chronic inflammation, macrophage activation, and resistance to apoptosis in ectopic lesions [61,62,63,64], but some studies have also suggested that IL-6 may limit the progression of endometriosis [63]. Although our study did not distinguish among specific cell populations expressing IL-6, both endothelial and stromal cells are likely to have contributed to the observed increase in IL-6 levels. In 2D cell culture, both cell types have been shown to synthesize IL-6 in response to pro-inflammatory cytokines [59,65,66].

In addition to IL-6, the pro-inflammatory chemokine IL-8 (CXCL8) was significantly and time-dependently expressed in serum co-cultures from patients with endometriosis. Elevated IL-8 levels have been widely reported across biological compartments in endometriosis, including peritoneal fluid, serum, and endometrial tissues [67,68,69], consistent with our findings. IL-8 expression has also been detected in monocultures of isolated endometrial stromal cells [70] and in ECs isolated from endometriotic lesions, indicating the contribution of these cells to systemic IL-8 levels in these patients [71]. IL-8 contributes to the chronic inflammatory process characteristic of endometriosis by promoting immune cell recruitment and establishing a microenvironment that supports the survival and growth of ectopic lesions [70,71,72,73].

The serum of patients with endometriosis also induced elevated IL-1β levels in the co-cultures. This expression increased after 48 h, showing a 6.6-fold increase over the levels found in endometriotic serum. Cells secrete IL-1β in response to inflammation. IL-1β acts as a potent signal, orchestrating immune responses and attracting immune cells to sites of infection or tissue damage. As a key regulator of innate immunity, IL-1β amplifies, inflammation by promoting the production of additional cytokines [74,75]. Thus, our findings suggest that co-cultures are highly responsive to their microenvironment, which caused a marked increase in IL-1β secretion under inflammatory conditions. The IL-1β expression in co-cultures was different from the later expression patterns (10 days of culture) previously reported in endometrial stromal and epithelial cell co-cultures [75,76]. Signaling between endothelial and stromal cells may have interfered with this early production, a mechanism that warrants further investigation. While this study did not explore the causal relationships among IL-1β, IL-6, and IL-8, the elevated levels of these cytokines across various endometriosis models [61,77,78,79] indicates their significance.

In summary, our results showed that this organoid-like 3D endometrial co-culture is a valuable tool for studying endometrial physiology and pathology. The interface between the endometrial tissue and endothelium is not just a passive physical barrier but a dynamic structure with multiple functions encompassing endocrine, metabolic, immunological, and enhanced-surveillance roles that may improve our understanding of the relationships between tissue and circulating factors. When combined with high-throughput analysis of biological molecules (DNA, RNA, proteins, and metabolites), this model can help address knowledge gaps in endometrial function and dynamics.

**Limitations of the study:** The main limitation of the present study was the reliance on human-derived material. While these samples offered advantages over animal models or immortalized cells, they had inherent individual variability that could not be fully controlled. Furthermore, primary cells tend to undergo senescence over time, whereas immortalized cell lines are designed to maintain consistent growth. Nevertheless, one advantage of the primary cell model is the possibility of maintaining cells from the same patient obtained from multiple culture passages, allowing their use in diverse assays and potentially supporting personalized therapeutic testing.

Moreover, the literature indicates that the use of Matrigel and collagenous matrices as scaffolds for endothelial or stromal cells presents certain limitations [80,81]. While Matrigel may contain many extraneous signaling molecules that could drown out the signals produced by the cells it supports, only experiments with Matrigel can yield consistent results in spread out and total coverage of the stroma compartment by ECs. In addition, collagen matrices have been reported to exhibit significant shrinkage and retraction over the course of a culture [82,83,84]. Notably, in our study, we did not observe matrix shrinkage in the upper compartment of the Transwell or in slide tests. This may be due to the limited experimental time (maximum, 96 h). Furthermore, our matrices contained hyaluronic acid (HA), which may have stabilized the collagen structure. HA may have served as a hydrating factor for the collagen gel or may have been associated with proteoglycans physically linked to collagen fibrils [85,86], increasing the mechanical stability of the network.

The stromal microenvironment is composed of many different cell populations, but this study only considered stromal cells to accommodate our study design. Although the model does not fully recreate the complex architecture of the native endometrium, it may sufficiently recapitulate the endothelial–stromal interface in vitro, enabling the investigation of cell–cell and ECM–cell interactions. Future studies should aim to include epithelial and immune cells as well as omics analysis of the cell populations.

Finally, the enrollment of patients with endometriosis did not account for general population demographics, such as socioeconomic status, ethnicity, disease severity, or disease duration. Although heterogeneous serum was used to demonstrate the co-culture’s potential to respond to environmental challenges, this study did not specifically examine how disease status influences gene expression or other cell behaviors. Nevertheless, co-cultures could be employed in future studies with appropriate power analyses to investigate endometriosis in greater detail.

## 4. Materials and Methods

### 4.1. Study Design

This experimental study aimed to demonstrate the feasibility of a 3D co-culture system mimicking the endometrial endothelium–stroma interface, with the primary objective of developing a functional in vitro model to study the endometrial biology and pathology. Cells isolated from endometrial biopsies were characterized to confirm their identity, viability, and retention of cell properties. The 3D model was constructed by embedding stromal cells in a collagenous matrix and layering endothelial cells to recreate the interface. To test its functional relevance, the model was exposed to serum from patients with an inflammatory condition (endometriosis), and its response was evaluated.

### 4.2. Participants

The Research Ethics Committee at the Institute of Biomedical Sciences, University of São Paulo, approved the protocol for human participants (approval numbers: 692.457 and 6.342.104), and the study was registered with Plataforma Brasil (numbers: 14978613.3.0000.5467, 14 June 2014 and 72592823.0.0000.5467, 9 October 2023). Written informed consent was obtained from all participants.

Blood samples were collected from women with endometriosis (*n* = 5) at the Hospital das Clínicas, School of Medicine, University of São Paulo. Endometriosis was diagnosed on the basis of the results of transvaginal ultrasonography after bowel preparation [87] and serum cancer antigen 125 (CA125) levels above 35 U/mL. In all cases, the diagnosis and staging of endometriosis were subsequently confirmed by laparoscopy.

Endometrial tissue (*n* = 13) and blood samples (*n* = 5) obtained from young healthy oocyte donors at Huntington Medicina Reprodutiva in São Paulo, Brazil, without clinical or ultrasound evidence of endometriosis, served as the control group. These participants were evaluated by clinical examination and transvaginal ultrasonography as part of the standard screening process for oocyte donation. Laparoscopy was not performed in this group, as it would not be ethically justified in asymptomatic healthy donors, and CA125 levels were not routinely assessed.

The inclusion criteria for all participants were as follows: age ≤ 35 years, body mass index (BMI) ≤ 30 kg/m^2^, both ovaries present, absence of clinically significant pelvic or uterine anomalies, and basal serum hormone levels within the normal reference ranges (follicle-stimulating hormone [FSH], ≤12 IU/L; luteinizing hormone [LH], ≤9 IU/L; estradiol [E2], 70–500 pmol/L) during the follicular phase. The exclusion criteria included the presence of chronic, degenerative, genetic, or autoimmune diseases; known infections such as human immune virus (HIV), human hepatitis C virus (HCV), or hepatitis B virus (HBV); confirmed abuse of drugs or any other substances during the treatment period; diagnosis of hydrosalpinx or adenomyosis; uterine malformations; or a history of uterine surgery (such as myomectomy or curettage). The demographic and clinical characteristics of the patients are summarized in Table S1.

### 4.3. Sample Collection

#### 4.3.1. Endometrial Biopsy

Endometrial biopsies were collected from women without endometriosis (*n* = 13), all of whom were young oocyte donors undergoing standard ovarian stimulation, as previously described by Domingues et al. [88], before oocyte retrieval (Figure 1A). Following cervical antisepsis, endometrial fragments were collected using a biopsy catheter (Pipelle de Cornier; Prodimed, Neuilly-en-Thelle, France). The endometrial thickness was not assessed at the time of biopsy, because the procedure was focused on oocyte retrieval, rather than endometrial preparation or embryo transfer. The samples were immediately transferred to DMEM/F12 (Sigma-Aldrich, St. Louis, MO, USA) and maintained at 4 °C for up to 1 h before processing.

#### 4.3.2. Blood Collection

Blood samples were collected from the control group (C, *n* = 5), which was defined as patients without endometriosis, before endometrial biopsy. Samples from the endometriosis group (E, *n* = 5), which consisted of patients with endometriosis (characteristics described in the Section 4.2) who were not receiving hormonal treatments (treatment was interrupted for 1–2 months before the surgical removal of endometriosis foci), were collected during their regular outpatient appointments at the end of the follicular phase and the beginning of the ovulatory phase of the menstrual cycle (Table S1).

All collections were conducted in the morning after an overnight fast. Peripheral blood samples (10 mL per patient) were collected in serum separator tubes without anticoagulants, in accordance with standard clinical protocols and ethical guidelines. After clot retraction for 1 h at room temperature (18–25 °C), the samples were centrifuged at 1500× *g* for 10 min, and the supernatants were collected. To minimize individual variability, including variability in age and other factors associated with endometriosis, sera from controls and patients were pooled separately and stored in aliquots at −80 °C for subsequent culture assays.

### 4.4. Cell Dissociation and Initial Cell Seeding

The biopsy samples were washed with calcium- and magnesium-free Hank’s balanced salt solution (HBSS) and then dissociated with a scalpel. The tissues were subsequently digested with collagenase II (1 mg/mL) and DNase I (0.1 mg/mL), both from Sigma-Aldrich (Figure 1B).

After digestion, the cells were filtered through a 70-µm Cell Strainer (BD Biosciences^®^, Franklin Lakes, NJ, USA) to separate endometrial glands (Figure 1C). The cell suspension was centrifuged (1000× *g*, 10 min, 4 °C) and resuspended in DMEM/F12 (Sigma-Aldrich) with 20% human serum (HS, from healthy patients) to isolate stromal cells (fibroblasts and decidual cells) and endothelial cells (ECs). Erythrocytes were removed by brief incubation with ammonium chloride solution (5 min, 25 °C), followed by a second centrifugation.

ECs were isolated by positive selection using a MACS immunomagnetic column with microbeads coupled to a primary anti-CD105 antibody (endothelial compartment; Miltenyi Biotec, Auburn, CA, USA). This step was performed in accordance with the manufacturer’s instructions (Figure 1D). Adherent cells (Figure 1E) were removed and resuspended in a supplemented DMEM/F12 medium (20% HS, 0.05 mg/mL sodium pyruvate, 0.5 mg/mL calcium lactate, 1% nonessential amino acids, 0.2 µg/mL insulin, 4 mg/mL bovine serum albumin, and antibiotics [50 U/mL penicillin, 0.05 mg/mL streptomycin]). All supplements were obtained from Gibco^®^ Company (Life Technologies, Waltham, MA, USA).

Cells that passed through the column (fibroblasts and decidual cells) (Figure 1F) were collected and resuspended in the same medium (stromal compartment). Stromal cells and ECs (Figure 1G) were plated separately and cultured in DMEM/F12 with supplements at 37 °C in a humidified 5% CO_2_ atmosphere. ECs were plated on 1% gelatin (Sigma-Aldrich). The cells underwent 3–5 passages, were dissociated with 0.025% trypsin/0.02% ethylenediaminetetraacetic acid (EDTA; Adolfo Lutz, São Paulo, SP, Brazil) and were then frozen in 10% dimethyl sulfoxide (DMSO, Sigma-Aldrich) in HS until 3D co-culture preparation. Each passage was characterized by immunohistochemical and flow cytometric analyses.

### 4.5. Subtyping of the Endometrial Cells

#### 4.5.1. Cell Proliferation

Freshly harvested cells and those cultured for 3 and 5 passages were plated in 24-well plates (Corning, São Paulo, SP, Brazil) at a density of 1 × 10^4^ viable cells/mL. The cells were collected and counted at intervals of 24 h. The Countess™ automated cell counter (Invitrogen, Thermo Fisher Scientific, Waltham, MA, USA) was used for cell counting after staining with 0.4% trypan blue (Sigma-Aldrich) to exclude dead cells.

#### 4.5.2. Immunohistochemical Analyses

Cells cultured in monolayers were washed in phosphate-buffered saline (PBS) and fixed in methanol at −20 °C for 24 h. The cellular immunofluorescence was then evaluated. Primary antibodies (sources and targets listed in Table S3) were diluted in 0.05% fish skin gelatin (Sigma-Aldrich) in PBS (1:50) and incubated for 1 h at room temperature. Subsequently, the cells were incubated with fluorescein isothiocyanate (FITC)- or rhodamine-conjugated secondary antibodies for an additional 1 h (details presented in Table S3).

Sections were mounted using 4′,6-diamidino-2-phenylindole (DAPI) Vectashield mounting medium (Vector Laboratories, Burlingame, CA, USA) and analyzed using an Axioskop2 fluorescence microscope (Carl Zeiss, Mannheim, Germany). Reactions performed without incubation with the primary antibodies were treated as controls. Reactivity to each antibody was estimated as the percentage of reactive cells among the total population of DAPI+ nuclei, counted in six distinct randomly selected microscopic fields (×40 magnification) from three cultures performed on different occasions and passages.

#### 4.5.3. Flow Cytometry for Phenotyping of Dissociated Cells

The cultured cells were analyzed by flow cytometry using the same primary antibodies to assess their purity (Table S3). To prepare for the analysis, the cells were detached from the culture flask with 0.025% trypsin/0.02% EDTA (Adolfo Lutz), centrifuged at 800× *g* (4 °C) for 10 min, counted, washed, and resuspended in PBS.

Samples were fixed with the Fix & Perm kit (Cytofix Fixation Buffer, cat. #554714; BD Biosciences, San Diego, CA, USA). They were incubated with the primary antibodies for 30 min at room temperature (sources and dilutions in Table S3), followed by incubation with the secondary antibodies for 30 min in the dark (see Table S3), along with a cell death marker conjugated to V500 (Fixable Viability Stain 510, Cat. #564406; BD Biosciences). Data were acquired using a BD Canto (BD Biosciences) with FACS Diva software version 8.0 (Becton Dickinson, Franklin Lakes, NJ, USA), which collected the data for 10,000 events using a general gate based on forward scatter (FSC) and side scatter (SSC) parameters. FlowJo software version 10 (BD Biosciences, Ashland, OR, USA) was used for analysis.

### 4.6. Constructing the 3D Co-Culture System

The ECM support medium (Figure 1H) consisted of fibronectin and collagen V combined (0.04 μg/mL), collagen I and collagen III (320 μg/mL each), and hyaluronic acid (300 μg/mL). All ECM components obtained from BD Biosciences were prepared in accordance with a protocol adapted from the study by Ramshaw et al. [39]. The mixed ECM components were stabilized on ice by adding 10 µL of 0.1 M NaOH. Separately, 1 × 10^5^ stromal cells were resuspended in 30 µL of supplemented culture medium. The cell suspension was then combined with the prepared ECM mixture at a 1:6 (*v*/*v*) ratio, yielding a total volume of 180 µL.

The solution was then pipetted into the upper chamber of a Transwell (pore size, 8.0 µm; Corning, NY, USA), placed in a 24-well plate, and incubated for 30 min at 37 °C under sterile conditions to allow collagen polymerization. Subsequently, the supplemented DMEM/F12 medium was added to the upper and lower chambers of the well, and the plate was incubated for 24 h (Figure 1I,J).

After gel polymerization, the medium in the upper chamber was carefully removed. A Matrigel (BD Biosciences) bath (Figure 1K), prepared at a 1:2 ratio in supplemented DMEM/F12 medium, was applied to the surface of the gelled matrix containing the cells (15 μL per insert, gently spread over the gel surface). After Matrigel polymerization (1 h at 37 °C), 5 × 10^4^ ECs suspended in 250 μL of supplemented DMEM/F12 medium (Sigma^®^-Aldrich) were added to the upper chamber (Figure 1L). The co-culture system was then maintained in an incubator at 37 °C with 5% CO_2_ for 24–48 h. Stromal and ECs from the third and fifth passages were tested for construction of the 3D model.

### 4.7. Morphological Analysis and Tissue Viability

Tissue viability was assessed by measuring the release of lactate dehydrogenase (LDH) into the incubation medium at set time points. LDH concentrations were determined using a Lactate Dehydrogenase Assay kit (Sigma-Aldrich) in accordance with the manufacturer’s instructions.

The morphology of the 48 h co-cultures (48 h after EC cell seeding) was assessed using paraffin-embedded sections, immunofluorescence analyses, and high-resolution imaging (Figure 1N). Transwell inserts were removed from the culture plate, washed with PBS, and embedded in HistoGel (HistoGel™; Thermo Scientific, Kalamazoo, MI, USA). After HistoGel solidification, the membrane support containing the cells and ECM was carefully excised. The released tissue was then fixed and processed for the analysis as required (as described below).

For light microscopy analyses, the tissues were first fixed for 1–2 h in PBS containing 2% paraformaldehyde (Sigma-Aldrich) and then routinely processed for paraffin embedding (Paraplast; Sigma-Aldrich). Five-micrometer-thick sections were then deparaffinized, hydrated, and used for hematoxylin and eosin (H&E) staining and immunofluorescence analyses to characterize the system.

For immunofluorescence analyses, the sections were incubated in PBS containing 3% glycine (Sigma-Aldrich) and 0.05% fish skin gelatin (Goldfish Gelatin; Sigma-Aldrich) for 1 h each to block nonspecific antigenic sites. The antibodies and protocols used in this step were the same as those employed in monolayer cultures to characterize stromal and ECs. Further details are provided in Table S3.

For ultrastructural analyses, the 3D co-culture tissues were fixed in 2% glutaraldehyde in 0.1 M sodium cacodylate buffer for 2 h, which was followed by post-fixation in 1% osmium tetroxide for 1 h. The samples were then embedded using the standard Spur resin procedure. All reagents were obtained from Polysciences Inc. (Warrington, PA, USA). Ultrathin sections were stained with uranyl acetate and lead citrate solutions and observed using a JEOL Transmission Electron Microscope (JEM-100CXII, Akishima, Tokyo, Japan) operating at 80 kV.

### 4.8. Challenging 3D-Co-Cultures with Inflammatory Sera

After model characterization, co-cultures were prepared using cells from five donors, which were divided into two subgroups (Figure 1M). One group was maintained with control serum (control group, C), and the other was treated with serum from women with endometriosis (endometriosis group, E). The sera were added to the co-culture system 24 h after seeding ECs, and the co-cultures were maintained under standard conditions for another 48 h. Each experiment was conducted in triplicate. After serum exposure, cells and supernatants were collected and processed for cytokine analysis.

#### Cytokine Evaluation

After 24 and 48 h of co-culture, the supernatants from all samples were collected, and the 3D cultures were washed several times with PBS. The samples were then centrifuged at 1000× *g* for 10 min at 4 °C, which was followed by two washes in cold PBS and another centrifugation. To prepare the homogenate, 50 µL of radioimmunoprecipitation assay (RIPA) buffer, 0.5 µL of protease inhibitor, and 0.25 µL of Na_3_VO_4_ were added to the cellular pellet. The mixture was centrifuged again at 2000× *g* for 10 min and stored at −80 °C until the cytometric assay was performed.

The cytometric bead array (CBA, cat#551811; BD Biosciences, San Diego, CA, USA) was used to measure the levels of interleukin (IL)-6 (sensitivity, 2.5 pg/mL), IL-8 (3.6 pg/mL), IL-12p70 (1.9 pg/mL), IL-10 (3.3 pg/mL), IL-1β (7.2 pg/mL), and tumor necrosis factor (TNF)-α (3.7 pg/mL) in both patient sera and co-culture samples (Figure 1N). The procedures were performed in accordance with the manufacturer’s instructions. The data were acquired using an LSR Fortessa X20 flow cytometer (BD Biosciences). The results were generated in both graphical and tabular formats using the BD CBA Analysis Software version 1.4 (BD Biosciences), calculated from a standard curve, and reported in pg/mL.

### 4.9. Statistical Analysis

For values that showed a normal distribution (confirmed by the Shapiro–Wilk test), the parametric distribution was analyzed using one-way analysis of variance (ANOVA), followed by Tukey’s or Dunnett’s test for multiple comparisons, where appropriate. For values showing an asymmetric distribution, the Kruskal–Wallis test was performed, along with Dunn’s post hoc test for multiple comparisons. To analyze the clinical and demographic characteristics of the patients, significance was assessed using ANOVA, which was followed by a post hoc Scheffe’s multiple-comparison test. Statistical analyses were performed using GraphPad Prism software (version 10.0; La Jolla, CA, USA). Statistical significance was set at *p*-value < 0.05.

## 5. Conclusions

Our findings suggest that this organoid-like co-culture model may facilitate evaluations of the influence of circulating components on the endometrial compartment through a functionally relevant endothelial–stroma interface, which is often underestimated. Potential investigations extending the model’s possibilities include studies of paracrine interactions between cells in these compartments, analyses of differential gene expression, and assessments of the secretory activity of this reconstituted interface under various physiological, medication-related, or stress conditions that may contribute to endometrial failure and infertility. Additionally, the possibility of individualized therapy involving different steps of the treatment and studies of the same patient’s cells in co-cultures along the course of the treatment are worth consideration. Overall, our results demonstrate that stromal cells–ECs can respond to environmental factors, indicating a promising strategy for elucidating how circulating molecules may influence endometrial tissue physiology and disrupt reproductive processes.

## Figures and Tables

**Table 1:** Cytokine levels measured in the serum of patients without endometriosis (Cp) and with endometriosis (Ep).

Cytokines	Cp	Ep	*p*
IL-12p-70	0.016 ± 0.011	0.54 ± 0.13	0.0205
IL-10	0.1 ± 0.04	0.012 ± 0.015	0.4595
IL-1β	8.09 ± 4.38	20.3 ± 6.45	0.4828
IL-6	4.68 ± 4.45	9.82 ± 6.30	0.0393
IL-8	8.82 ± 2.58	141.58 ± 32.26	0.0186
TNF-α	0.28 ± 0.19	0.26 ± 0.121	0.8870

Cytokines (pg/mL), median ± standard deviation; one-way ANOVA and Tukey’s multiple comparisons test (*n* = 3). Gray background color identify data below the assay sensitivity.

## Data Availability

The data that supports the findings of this study are available from the corresponding author [EB], upon reasonable request.

## Supplementary Materials

The following supporting information can be downloaded at https://www.mdpi.com/article/10.3390/ijms27135645/s1.

## Abbreviations

The following abbreviations are used in this manuscript:

3D	Three-dimensional
IVF	In Vitro Fertilization
Kg	Kilogram
Cm	Centimeter
vs.	Versus
BMI	Body Mass Index
N	Number
ECS	Endothelial cells
h	Hour
LDH	Lactate dehydrogenase
IGFBP1	Insulin-like Growth Factor Binding Protein 1
ECM	Extracellular Matrix
IL-6	Interleukin-6
IL-8	Interleukin-8
IL1β	Interleukin-1 beta
IL-2	Interleukin-2
IL-10	Interleukin-10
IL12p70	Interleukin-12 p70
TNF-α	Tumor Necrosis Factor alpha
DMEM/F12	Dulbecco’s Modified Eagle Medium/Nutrient Mixture F-12
H&E	Hematoxylin and Eosin
CBA	Cytometric Bead Array
IF	Immunofluorescence
FC	Flow Cytometry
VIM	Vimentin
vW	Von Willebrand factor
CK	Cytokeratin
DAPI	4′,6-diamidino-2-phenylindole
FITC	Fluorescein Isothiocyanate
2D	Two-dimensional
CXCL8	Motif Chemokine Ligand 8
HA	Hyaluronic Acid
FSH	Follicle-stimulating Hormone
LH	Luteinizing Hormone
HIV	Human Immune Virus
HCV	Human Hepatitis C virus
HBV	Hepatitis B Virus
E2	Estradiol
GnRH	Gonadotropin-releasing agonist
C	Control group
E	Endometriosis group
DMSO	Dimethyl Sulfoxide
PBS	Phosphate-buffered Saline
EDTA	Ethylenediaminetetraacetic acid
FSC	Forward Scatter
SSC	Side Scatter
Cp	Control patient (without endometriosis)
Ep	Patient with endometriosis
