# Supplementary material for: Three-Dimensional Organoid-like Co-Culture of Human Endometrial Endothelial and Stromal Cells to Study Endometriosis-Associated Responses
Source: Int J Mol Sci. 2026 Jun 23;27(13):5645. doi: 10.3390/ijms27135645 (PMC13360939; doi:10.3390/ijms27135645)
Supplement: Supplementary file 1 [file ijms-27-05645-s001.zip › ijms-4331355-supplementary.pdf]

Supplementary Figures

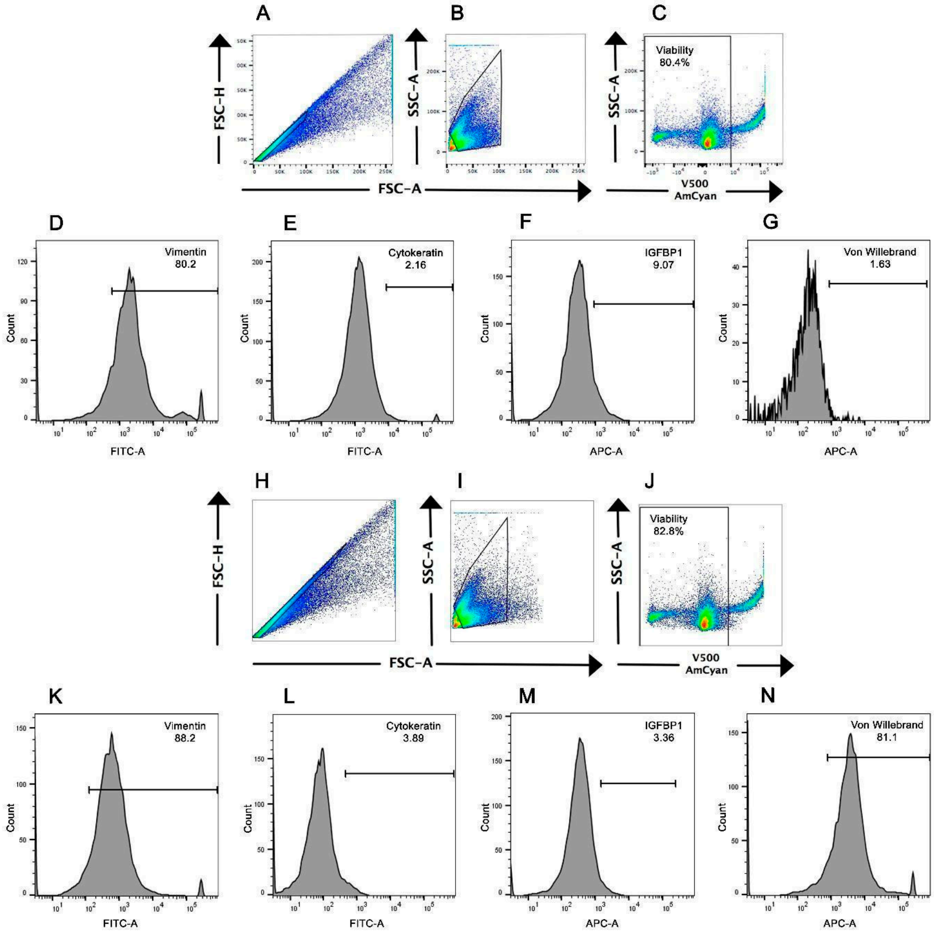

Figure S1. Characterization by flow cytometry of the cells negatively (stromal cells, A-G) and positively (endothelial cells, H-N) selected in the magnetic column. A–B; H–I: Exclusion of doublets and debris. **C, J:** Assessment of cell viability (80.4% and 82.8%, respectively for stromal and endothelial cells). **D, K:** Reactivity of stromal (**D,** 80.2%) and endothelial (**K**, 88.2%) cells to vimentin. Stromal cells were reactive to cytokeratin (**E,** 2.16 %), IGFBP1 (**F**, 9,07%) and Von Willebrand (1.63%). **E:** Reactivity of endothelial cells to cytokeratin is shown in **L** (3.89%), to IGFBP1 in **M** (3.36%), and to Von Willebrand in **N** (81.1%).

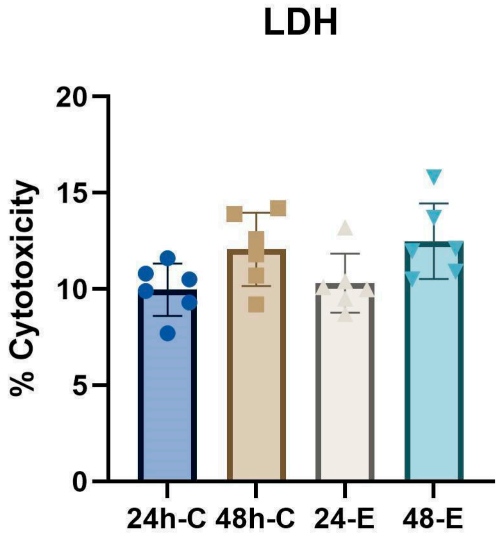

Figure S2. Concentration of lactate dehydrogenase (LDH) was analyzed in the supernatant of 3D-endometrial co-cultures. Samples were analyzed 24 and 48 h after the introduction of control (C) and endometriosis (E) serum from patients. Data are presented as mean ± SD. (ANOVA); n=3 independent experiments.

Supplementary Tables

Table S1. Clinical information about the patients.

	**Non-endometriosis**	**Endometriosis**
**Number of patients**	13	5
**Age (years)**	23.5 ± 3.04	31.6 ± 5.36*
**BMI**	23.60 ± 2.3	24.69 ± 4.26
**Follicular phase**	61.5% (8)	80% (4)
**Ovulatory phase**	23.1% (3)	20% (1)
**Luteal phase**	15.3% (2)	0
**Stage III**	Na	20% (1)
**Stage IV**	Na	80% (4)

Data are expressed as mean ± standard deviation.
Significance was determined with analysis of variance, followed by post hoc Scheffe's multiple comparison test, *$P<0.05$. Na: not applicable

Table S2. Cytokine[1] profile of homogenates (H) and supernatants (S) of 24- and 48-h co-cultures treated with (a) serum of women without endometriosis (C) and (b) with endometriosis (E).

Cytokines	C-H-24h	C-S-24h	C-H-48h	C-S-48h
**IL-1β**	13.79 ± 6.3	23.50±10.6	24.29 ± 12.8	11.70 ± 4.2
**IL-6**	1.70 ± 0.5	2.03 ± 0.8	4.03 ± 3.0	2.09 ± 0.7
**IL-8**	10.52 ± 2.7	12.90 ± 7,6	14.89 ± 4.6	11.24 ± 2.4
**b. Cytokines**	**E-H-24h**	**E-S-24h**	**E-H-48h**	**E-S-48h**
**IL-1β**	30.40 ± 4.5	24.56 ± 3.0	75.60 ± 23.9	134.31± 27.9#c
**IL-6**	2.43 ± 0.8	2.44 ± 0.9	14.74 ± 1.8*a	17.39 ± 1.8**d
**IL-8**	121.32 ± 20.8#	131.71 ± 28.5#	220.44 ± 14.9#b	245.61 ± 45.9#e

[1]Cytokine levels were compared with 24-hour co-cultures (indicated by letters) and their respective control samples (indicated by symbols), with measurements expressed in pg/mL and reported as median ± standard deviation. Statistical significance was evaluated using one-way ANOVA, followed by Tukey's multiple comparisons test (n=3). When comparing cytokine levels after 48 hours of incubation to those from 24-hour cultures, both homogenate and supernatant samples exhibited a marked increase in IL-6 and IL-8. In contrast, IL-1β levels increased exclusively in the supernatants at this time point. When compared with their respective controls, significantly elevated IL-1β concentrations were observed in the 48-hour supernatants, while IL-6 levels increased in both homogenates and supernatants. Notably, IL-8 levels were elevated at all time points and in both sample types. [a,d]*P*=0.0001; [b]*P*=0.0169; [c]*P*=0.0022; [e]*P*=0.0069; #*P*<0.0001, **P*=0.0115; ***P*=0.0015.

Table S3. Antibodies used for immunofluorescence reactions and flow cytometry

Cell type / Target protein	Primary / Secondary Antibody	Source	Dilution	
			IF*	FC**
**Epithelial cells** CK: Cytokeratin	Rat monoclonal anti-h Cytokeratin	Dako (#IR-053)	1:50	1:250
	Goat anti-rat FITC conjugated	Sigma-Aldrich (#F6258)	1:400	1:250
**Stromal cells** VIM: Vimentin	Rat monoclonal anti-h Vimentin	Sigma-Aldrich (#V-4630)	1:50	1:250
	Goat anti-rat FITC	Sigma-Aldrich (t#F6258)	1:400	1:250
**Decidual cells** IGFBP1: Insulin-like growth factor binding protein 1	Rabbit polyclonal anti-h IGFBP1	Abcam® (#ab111203)	1:50	1:250
	Goat anti-rabbit FITC conjugated	Sigma-Aldrich (#AP124F)	1:400	-----
	Donkey anti-Rabbit Alexa Fluor® 647	Abcam® (#ab150075)	-----	1:400
**Endothelial cells** vW: von Willebrand factor	Rabbit polyclonal anti-h von Willebrand factor	Dako (#A0082)	1:50	1:250
	Goat anti-rabbit FITC conjugated	Sigma-Aldrich (#AP124F)	1:250	-----
**Endothelial cells** CD-105: Endoglin	Rabbit polyclonal Anti-CD-105	ThermoFisher (#PAS-12511)	1:50	-----
	Goat anti-rabbit FITC conjugated	Sigma-Aldrich (#AP124F)	1:250	-----

Dako Corp. Carpinteria, CA, USA; Sigma-Aldrich, St Louis, MO, USA; Abcam® Inc., Cambridge, MA, USA; ThermoFisher Scientific, Rockford, IL, USA. h: human; IF: immunofluorescence; FC: flow cytometry; FITC: fluorescein isothiocyanate; */** antibody dilution: *0.05% skin fish gelatin in PBS; ** PBS-0.5% FBS
